# Local induction of regulatory T cells prevents inflammatory bone loss in ligature-induced experimental periodontitis in mice

**DOI:** 10.1038/s41598-022-09150-8

**Published:** 2022-03-23

**Authors:** Ashlee C. Greene, Mostafa Shehabeldin, Jin Gao, Stephen C. Balmert, Michelle Ratay, Charles Sfeir, Steven R. Little

**Affiliations:** 1 Department of Chemical and Petroleum Engineering, University of Pittsburgh, Pittsburgh, PA USA; 2 Department of Oral and Craniofacial Sciences, School of Dental Medicine, University of Pittsburgh, Pittsburgh, PA USA; 3 Department of Periodontics and Preventative Dentistry, School of Dental Medicine, University of Pittsburgh, Pittsburgh, PA USA; 4 Department of Dermatology, University of Pittsburgh, Pittsburgh, PA USA; 5 Center for Craniofacial Regeneration, University of Pittsburgh, Pittsburgh, PA USA; 6 Department of Bioengineering, University of Pittsburgh, Pittsburgh, PA USA; 7 McGowan Institute for Regenerative Medicine, University of Pittsburgh, Pittsburgh, PA USA; 8 Department of Immunology, University of Pittsburgh, Pittsburgh, PA USA; 9 Department of Ophthalmology, University of Pittsburgh, Pittsburgh, PA USA; 10 Department of Pharmaceutical Sciences, University of Pittsburgh, Pittsburgh, PA USA

**Keywords:** Drug delivery, Regulatory T cells, Biomedical engineering

## Abstract

Periodontitis (periodontal disease) is a highly prevalent disease, affecting over 65 million adults in the United States alone. Characterized by an overburden of invasive bacteria, gum inflammation and plaque buildup, over time, these symptoms can result in severe loss of gingival tissue attachment, bone resorption and even tooth loss. Although current treatments (local antibiotics and scaling and root planing procedures) target the bacterial dysbiosis, they do not address the underlying inflammatory imbalance in the periodontium. In the healthy steady state, the body naturally combats destructive, imbalanced inflammatory responses through regulatory pathways mediated by cells such as regulatory T cells (Tregs). Consequently, we hypothesized that local enrichment of regulatory lymphocytes (Tregs) could restore local, immunological homeostasis and prevent the main outcome of bone loss. Accordingly, we locally delivered a combination of TGFβ, Rapamycin, and IL2 microspheres in a ligature-induced murine periodontitis model. Herein, we have demonstrated this preventative treatment decreases alveolar bone loss, increases the local ratio of Tregs to T effector cells and changes the local microenvironment’s expression of inflammatory and regenerative markers. Ultimately, these Treg-inducing microspheres appear promising as a method to improve periodontitis outcomes and may be able to serve as a platform delivery system to treat other inflammatory diseases.

## Introduction

Periodontitis is a chronic inflammatory oral disease, with its most severe form impacting over 11% of individuals globally. In 2010, it was the sixth-most prevalent condition worldwide^1^. Traditionally, periodontal pathogens have been thought of as the primary agitators and initiators of disease with the overburden of these pathogens causing an accumulation of biofilm and plaque, loss of gingiva and periodontal ligament attachment, pocket formation and eventual bone and tooth loss^2^. Therefore, traditional periodontitis treatments target the local oral bacterial burden. Clinically, scaling and root planing (SRP) would be performed for the physical debridement and removal of bacterial plaque and calculus from the tooth and root surfaces^3^. The addition of a systemic or local adjunct antibiotic therapy may also administered. However, while an overburden of periodontal pathogens can initiate oral dysbiosis of the microbiome and is a primary causal factor, literature suggests the immune system also plays a prominent role; an unbalanced inflammatory response produces the severity of symptoms that leads to both hard and soft tissue destruction^4^.

In the healthy steady state, the body employs many natural immune regulatory mechanisms that restore unbalanced inflammatory responses back to homeostasis. A subset of T lymphocytes, regulatory T cells (Tregs), are professional, regulatory immune cells that function through both direct cell–cell contact and secreted signals^5^. Our previous studies^6^ have suggested that these cells can be locally recruited in the local periodontium environment, leading to the reestablishment of homeostasis in periodontal disease. Notably, endogenous Tregs exist as a relatively small percentage of lymphocyte populations in the periphery (5–10%)^7^, and therefore recruitment strategies are limited by the number of Tregs, and also could be less effective in patients with systemic diseases that may alter the prevalence of endogenous Tregs^8–10^.

We have also previously demonstrated that a combination of factors (IL2, TGFβ and Rapamycin) leads to the local induction of a highly prevalent population of CD4 + naïve T cells, differentiating them into Tregs at the site of administration in various models^11–15^. Notably, IL2 and TGFβ (in small quantities delivered locally) are cytokines known to promote the growth and differentiation of naïve T cells into regulatory T cells^16^. Rapamycin is a small molecule, mTorr inhibitor capable of preventing the differentiation of naïve T cells into other T-cell types^17^. Here, we hypothesize that the local delivery of a formulation with this same combination of factors could reduce inflammatory bone loss through Treg induction even in the ligature-induced model of periodontal disease. Accordingly, we have tested the efficacy of the local delivery (at the same time as disease induction) of a combination of TGFβ, rapamycin and IL2 encapsulated in degradable microspheres (TRI MPs) by assessing local Treg induction and key signs of disease amelioration in a preventive ligature-induced murine model for periodontal disease (see Supplementary Fig. S1). These formulations (as well as previously explored Treg recruitment strategies) may serve as a platform to treat a number of inflammation-based oral diseases.

## Results

### TRI MPs demonstrate morphology consistent with their 1-week release profiles

**Figure 1:**
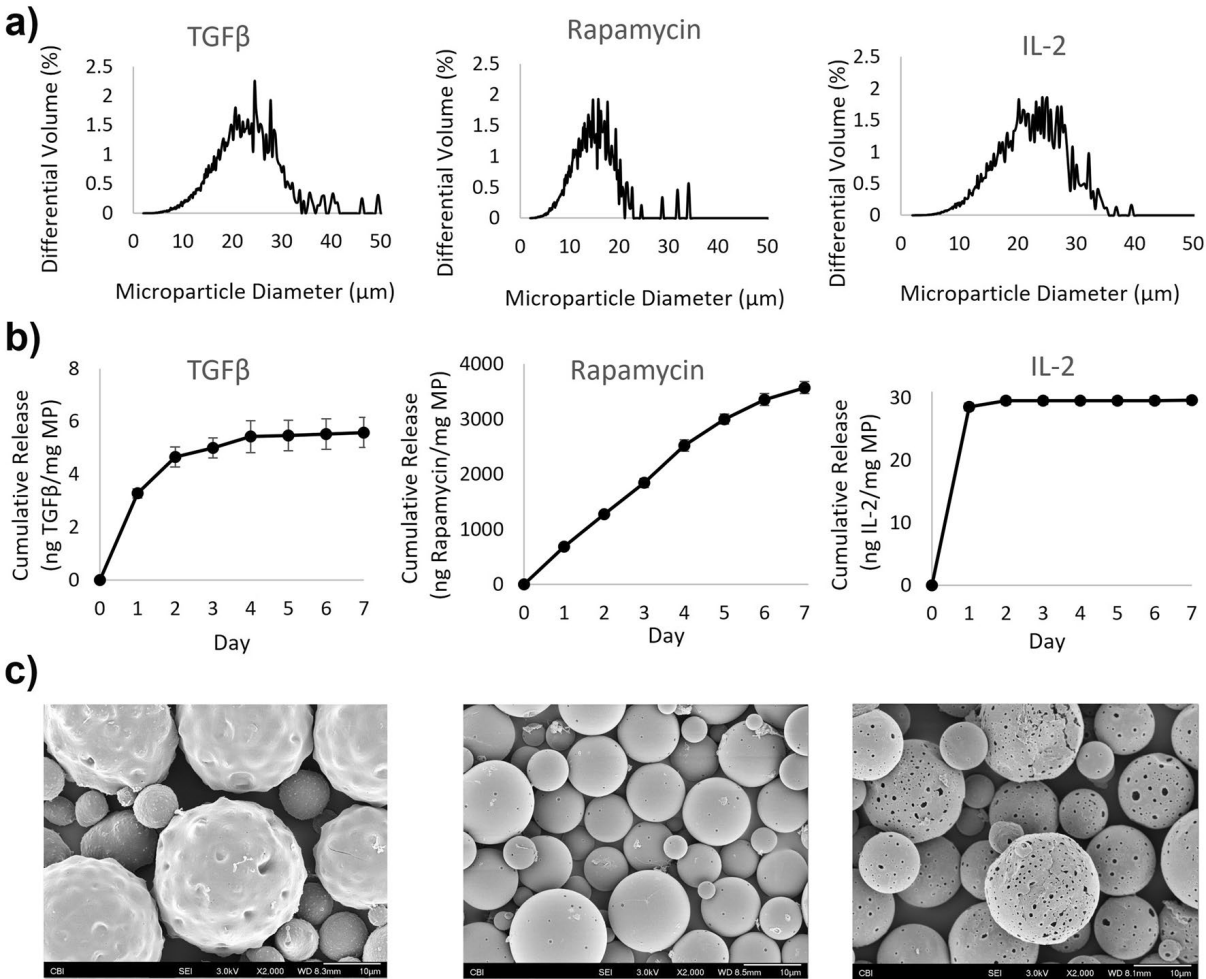
Characterization of the TRI (TGFβ, Rapamycin and IL-2) MPs. (**a**) Size distributions of microspheres (MPs) as measured by volume-impedance method with a Coulter Counter as mean ± standard deviation for TGFβ MPs (21.5 µm ± 6.8 µm), Rapamycin MPs (14.6 µm ± 6.8 µm), and IL-2 MPs (20.8 µm ± 6.1 µm). All Coulter Counter sigma counts were greater than 1000. (**b**) In-vitro release kinetics at 37 °C (n = 3) presented as the mean ± standard error of the mean for TGFβ MPs in PBS + 1%BSA, Rapamycin MPs in PBS + 0.2% Tween80, and IL-2 MPs in PBS + 1%BSA. (**c**) Representative scanning electron microscopy (SEM) images to observe surface morphology of TGFβ MPs, Rapamycin MPs, and IL-2 MPs at 2000x (scale bar = 10 µm).

Microspheres were first measured for size by volume-impedance method with a Coulter counter. Volume-average diameters were: TGFβ MPs (21.5 µm ± 6.8 µm), Rapamycin MPs (14.6 µm ± 6.8 µm), and IL2 MPs (20.8 µm ± 6.1 µm) (Fig. 1a). After sizing, release was assessed for 7 days to span the length of the ligature model timeline. For TGFβ MPs, approximately 5.5 ng were released before the profile reaches a plateau as compared to IL2 MPs with a burst release of approximately 28 ng before plateauing. Rapamycin MPs have a steadier, more linear release profile, reaching approximately 3500 ng by day 7 (Fig. 1b). Lastly, surface morphology was detected with scanning electron microscopy (SEM). As demonstrated in the representative SEM images, TGFβ MPs with the addition of poly (ethylene glycol) (PEG)-PLGA di-block copolymer, are spherical with an uneven or bumpy surface. Rapamycin MPs have a smooth and spherical appearance. IL2 MPs have a porous surface morphology (Fig. 1c).

### TRI MPs reduce alveolar bone resorption in an aggressive periodontitis model

**Figure 2:**
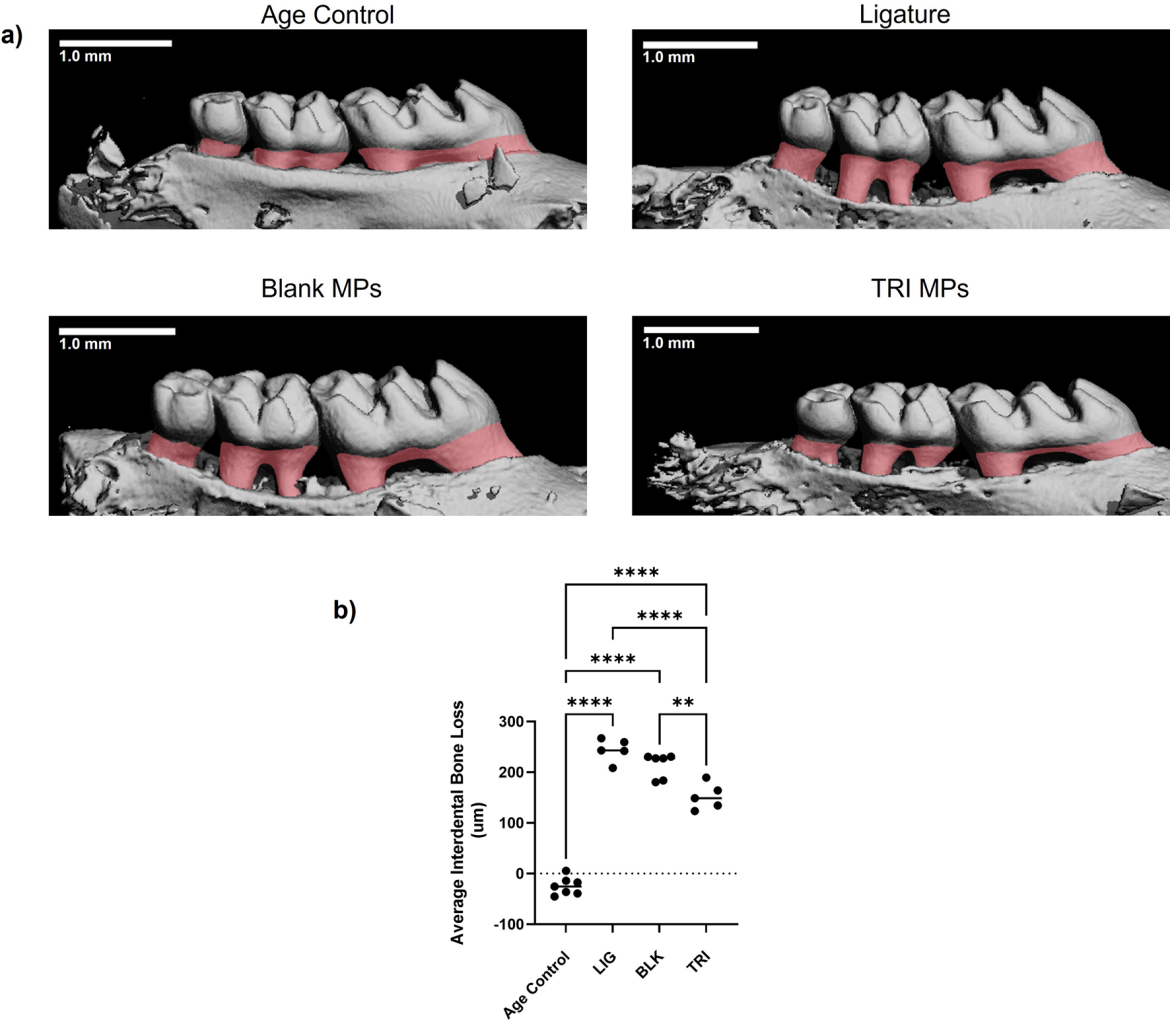
TRI MPs reduce ligature-induced alveolar bone resorption. An injection of MPs was administered to male Balb/c mice (6–8 weeks old) at the same time as ligature placement around the maxillary second molar. After 7 days, bone loss was assessed by micro-CT. (**a**) Representative 3D reconstructed micro-CT scans of the age control, ligature (diseased untreated), blank MPs (PLGA vehicle-only control) and TRI MPs experimental groups (scale bar = 1 mm). (**b**) Average bone loss quantified by linear interdental measurements between the cementoenamel junction and alveolar bone crest (CEJ-ABC) of the ligated maxillary second molar for each experimental group (n = 5–7 mice). A One-way ANOVA was performed followed by Tukey post-hoc analysis to compare the mean of every group with the mean of every other group to determine statistical significance where; **p ≤ 0.01, ***p ≤ 0.001, ****p ≤ 0.0001.

Next, local treatment with TRI MPs was evaluated in regard to the impact on alveolar bone loss in an aggressive, ligature-induced periodontitis model. Interdental bone loss was assessed for age control, diseased untreated (ligature only), vehicle control (ligature + blank MPs) and treated (ligature + TRI MPs) groups. Representative 3D reconstructed images reveal a visual difference (overlayed in red) in the bone level between the groups (Fig. 2a). Blinded analysis of the interdental measurements show our TRI MPs successfully reduced interdental alveolar bone loss by approximately ~ 30–40% when compared to the ligature only group and ~ 10–20% when compared to the vehicle control (Fig. 2b). Additionally, the age control group represents a baseline of very minimal natural bone loss with values near zero (or negative) from normalizing the left interdental measurements to the right side, internally. This is further validated with Masson–Goldner trichrome immunohistochemical staining. Lines of demarcation highlight the anatomical limits of the area where the focus is on the alveolar bone, gingiva and periodontal ligament. Arrows (Fig. 4a) point to areas of high bone loss for the ligature only and blank MPs group while arrows highlight areas of bone preservation in the age control and TRI MP group. The boxed region of the gingiva where the ligatures were applied along with asterisks at the periodontal ligament highlight clear differences in staining that is representative of changes in cell infiltrate and tissue health (Fig. 4a,b).

### Immunofluorescence suggests Treg induction capability of TRI treatment

**Figure 3:**
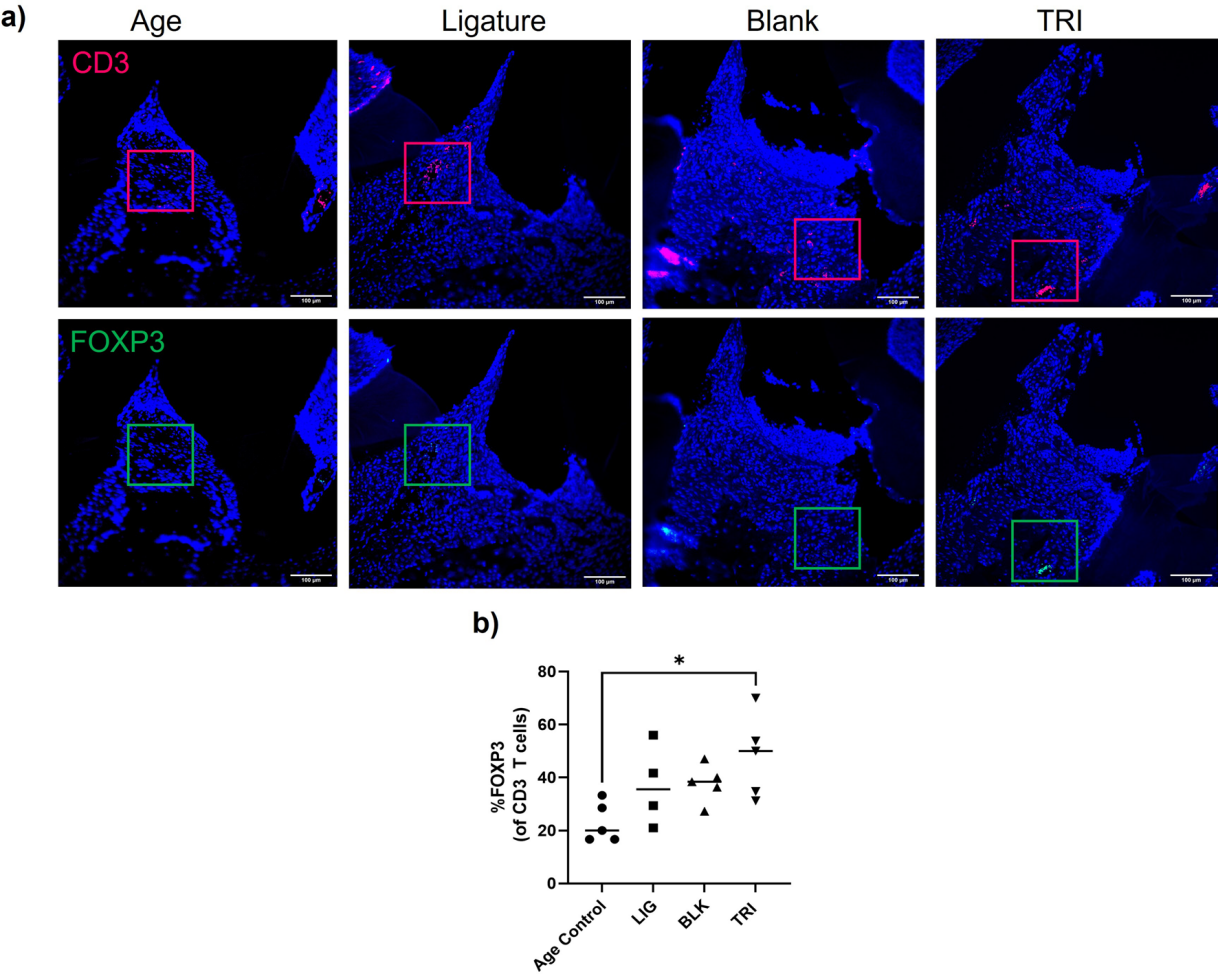
TRI MPs increase the local ratio of Tregs(FOXP3 positive cells). (**a**) Representative immunofluorescence staining for CD3 (total T cells), FOXP3 (Tregs), and DAPI (nuclei). Scale bars are 100 µm. (**b**) Blinded quantification of the %FOXP3 of CD3 total T cells (n = 4–5 per group) where a One-way ANOVA was performed followed by Tukey post-hoc analysis; *p ≤ 0.05.

In order to determine the Treg induction capability of the TRI MPs, total T cells (CD3 positive cells) and Tregs (FOXP3 positive cells) were stained by immunofluorescence. A blinded quantification of the percent Tregs [FOXP3 positive cells of total T cells (CD3 positive cells)] (representative images Fig. 3a) revealed an increase in this ratio when TRI MPs were administered (Fig. 3b). While there is only approximately 23.1% Tregs for the age control, there is 37.0% and 37.8% Tregs for the ligature and blank groups, respectively. The average percentage of Tregs for the TRI group was further increased to 48%, albeit only statistically significant when compared to the age control.

### Expression of pro-inflammatory versus pro-regenerative markers varied following treatment

Changes in gene expression of local markers in response to ligature administration and TRI MP treatment were also assessed. The expression of *Tnfsf11*, *Ncf1*,* Ifnγ,* and *Il4* was examined to capture the local pro-inflammatory context (Fig. 5a). Concurrently, the expression of *Tgfβ, Il10, Col1a1*, and* Timp1* was examined to capture the local pro-regenerative context (Fig. 5b). An upregulation in expression of pro-inflammatory markers was observed with the ligature group, blank group or both for *Ifnγ*, *Ncf1* and *Tnfsf11,* respectively. There was also a trend toward an increase in pro-inflammatory *Il4* for the ligature group, although not significant. For the anti-inflammatory markers, *Tgfβ* and *Il10* expression was upregulated for all groups that had received ligatures regardless of the treatment. Furthermore, while there was more of a bias towards upregulation of *Col1a1* and *Timp1* in the blank MP and TRI MP treatment groups, the difference between these two groups was surprisingly minimal.

## Discussion

Periodontitis is a common but complex oral disease that has historically been characterized by the presence of a bacterial assault^18,19^. Yet, in recent years, literature has elucidated a more nuanced but critical interplay between oral bacteria and the host response. Specifically, it is not just the microbial dysbiosis but an underlying unbalanced host immune response that is ultimately responsible for the inflammatory destruction and disease progression of periodontitis^20,21^. This renewed understanding has made way for a multitude of new immunomodulatory approaches as potential treatments; from the use of pro-resolving molecules like lipoxins and resolvins^22^ to strategies of modulating innate immune cells such as macrophages^23^ and cytokine signaling such as Del-1 or anti-IL17^24,25^. Therefore, in this study, we evaluated the effect of re-balancing the host immune response through an immune cell modulation strategy of locally inducing regulatory T cells (Tregs) for its impact on inflammatory bone loss in periodontitis.

Regulatory T cells (Tregs) are naturally positioned to counteract immune imbalance and inflammatory destruction^26^. Specifically, Tregs are known to accumulate in gingival tissues during flare-ups of periodontal disease in both human and animal models, emerging as key natural regulators of local inflammation^27^. Unlike strategies that completely block inflammation which can have potentially detrimental pathogenic effects (like systemic infections), Tregs naturally balance inflammatory signaling. Previously, we demonstrated the modulatory potential of Tregs in periodontitis through the local enrichment of Tregs using a *recruitment* strategy in a murine oral gavage model^6^. When Tregs were locally recruited and enriched such that the ratio of Treg to effector T cells favored resolution of inflammation, bone loss was reduced and there was a microenvironment shift towards regeneration. Furthermore, this strategy of harnessing the immunomodulatory power of Tregs did not have a negative pathogenic effect; it did not lead to an increase in total bacterial counts. Additionally, several mechanistic studies have also shown disease improvement could be disabled upon Treg inhibition. A reduction in Treg to effector T cell ratios due to the depletion of Tregs instead enabled further disease progression^6,28,29^. Therefore, while the potent ability of Tregs to counteract inflammation in periodontitis has been established, the efficacy of a Treg recruitment strategy is ultimately constrained by the pre-existence of local Tregs in sufficient numbers and ratio for recruitment.

A Treg *induction* strategy was developed to serve as an alternative (or potentially even an option to accompany) the Treg recruitment strategy. This induction strategy enables flexibility to increase the abundance of local Tregs through the induced differentiation of naïve T cells. As demonstrated previously^11^, a combination of TGFβ, rapamycin and IL2 encapsulated microspheres (TRI MPs) were utilized; known for their roles in the development and differentiation of Tregs, ability to decrease effector T cell populations and as a T cell proliferative agent, respectively. Although we have previously shown the ability of these factors (combined and not the factors individually) to induce Tregs from naïve T cells in-vitro^11^, and have demonstrated the resulting in-vivo Treg-mediated effects in the context other disease models^12–15^, this is the first time that this same induction strategy (TRI MP treatment) has been used in the context of periodontitis. Accordingly, we investigated the effect of this Treg induction strategy (with TRI MP treatment) on alveolar bone resorption. While there is no singular, ideal in-vivo animal model for murine induction of periodontitis and bone resorption, for this study, we utilized the ligature-induced murine model. Unlike the oral gavage model that has a long timeline for disease establishment and is based on the ability of the administered periodontal pathogen inoculation to take hold and locally colonize, the ligature-induced periodontitis model is advantageous in that disease can be initiated at a known time with a predictable, albeit more aggressive, disease propagation time course (when compared to the oral gavage model) of just a few days^30^. Therefore, the benefits of the ligature-induced model provide an optimal yet aggressive testing condition to rigorously evaluate the upper limits of the immunomodulatory capabilities of TRI MP treatment on periodontitis disease symptoms. On Day 0 (Supplemental Fig. S1), mice either received no ligatures (age control), or received ligatures without treatment (ligature only), received ligatures with blank MPs injection (vehicle control) or received ligatures with a TRI injection (consisting of TGFβ, Rapamycin and IL2 microspheres delivered together) since previous studies^12,13,15^ utilizing TRI MPs demonstrated the effect with all three factors was greater than any single factor or double factor combination. Herein, the ‘TRI’ formulation was adapted for use in the 7-day ligature-induced periodontitis murine model and the in-vitro results were consistent with our previous findings of characterization profiles that have demonstrated successful Treg induction (Fig. 1)^12–15^. Importantly, TRI MPs were able to demonstrate significant bone loss reduction when compared to the ligature group by approximately 30% (Fig. 2b). Unexpectedly, there was also a reduction in bone loss with Blank MPs (albeit to a lesser degree) when compared to the ligature group, by about 10–20%. Although this seemingly protective effect from the Blank MPs may not have been anticipated, this effect has actually been reported in other disease model applications of TRI MPs in the past^12,14^ and by others in the literature^31–33^. These findings may be due to PLGA-derived lactic acid and it’s latent immunomodulatory properties at the local site of administration^34^. Nevertheless, our studies show that TRI MP treatment reduced bone loss even in an extremely acute model of periodontitis.

Upon proof-of-principle demonstration of TRI MP treatment reducing inflammation-driven bone loss, we next wanted to investigate the direct effects of the TRI MP treatment on in situ Treg induction. Specifically, as observed in other disease models (allergic contact dermatitis, dry eye disease, transplant) with TRI MP treatment, it is not the number of induced Tregs but the resulting bolstered ratio of Tregs to effector T cells in the local environment, that enables the regulation of inflammatory signaling that leads to disease improvement^12,13,15,35,36^. This same principle has also been demonstrated periodontitis^37,38^. For this reason, we quantitatively assessed the ratio of Tregs to effector T cells after TRI MP administration in the ligature-induced periodontitis model. In the blinded quantification of immunofluorescence staining for FOXP3 (Tregs) and CD3 (total T cells), there was an increase in the ratio of Tregs to CD3 cells across all groups with ligature placement (Fig. 3b). This effect could be driven in part by the model itself; the presence of ligatures is supposed to increase inflammation as compared to the age control group, and this general increase in inflammation can increase the overall presence of responding lymphocytes (including Tregs) in the local area^39^. Yet, the TRI MP group still demonstrated the highest ratio of all measured groups, on average ~ 48%. This promising Treg induction by TRI MP lays the groundwork for future studies where we can even further bolster this ratio and explore its effects.

**Figure 4:**
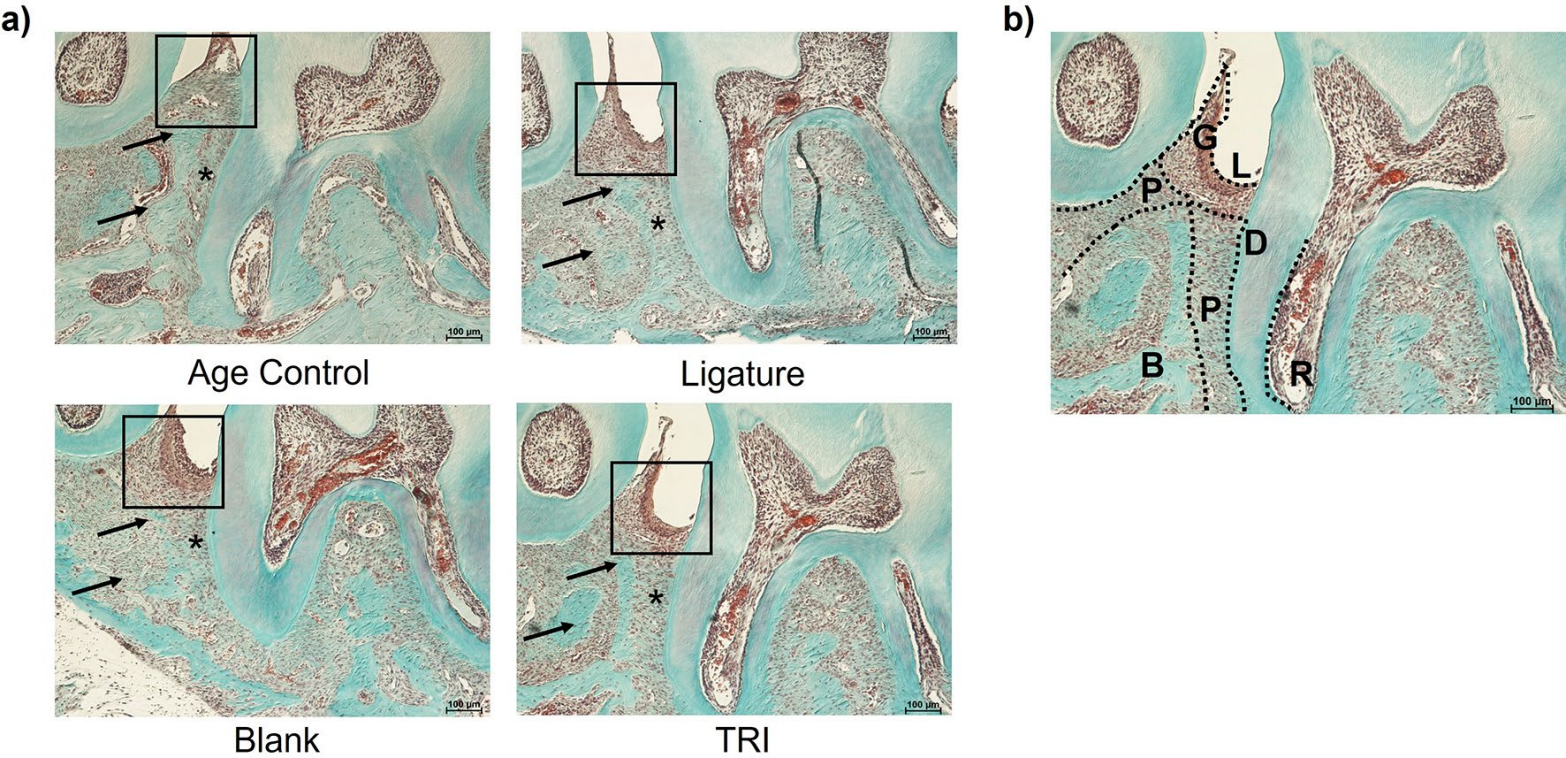
Representative immunohistochemistry of tissue sections near the ligated second maxillary molar. Maxilla from each group were paraffin embedded, cut into sagittal sections and stained to highlight anatomical changes due to disease induction and treatment. (**a**) Masson–Goldner trichrome immunohistochemical staining. Arrows point to regions of differences in alveolar bone structure, while a box and asterisks highlight the differences in cell infiltrate for the gingiva tissue and periodontal ligament (respectively). Scale bars are 100 µm. (**b**) Representative labeled Masson–Goldner stained sagittal section where anatomical limits are outlined for G; gingiva, L; ligature demarcation, D; dentin, P; periodontal ligament, B; alveolar bone and R; root.

**Figure 5:**
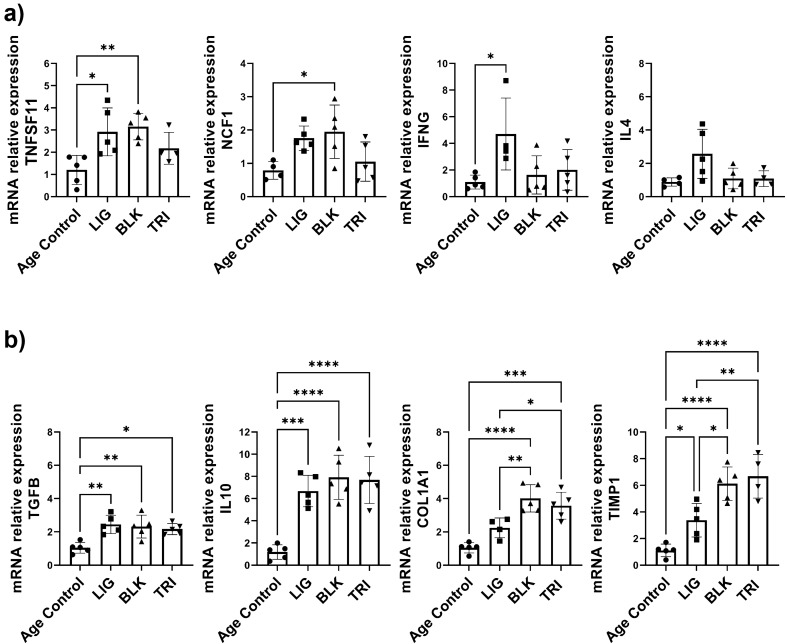
Expression of pro-inflammatory vs. pro-regenerative markers following treatment. Balb/c mice from Group A—ligatures applied with a TRI microsphere injection (TRI), Group B—ligatures applied with blank PLGA (vehicle only) microsphere injection (BLK), Group C—ligatures applied only (LIG) or Group D—no ligatures applied (age control) were sacrificed after 7 days. Periodontal gingival tissues were resected and mRNA expression was assed by qPCR for (**a**) markers indicative of a pro-inflammatory environment (*Tnfsf11, Ncf1, Ifnγ,* and *Il4*) versus (**b**) anti-inflammatory and pro-regenerative markers (*Tgfβ, Il10, Col1a1,* and *Timp1*). mRNA expression levels were compared by the value of 2^(−ΔΔCt)^ with *Gapdh* as the endogenous control. A One-way ANOVA was performed followed by Tukey post-hoc analysis to compare the mean of every group with the mean of every other group to determine statistical significance where; **p ≤ 0.01, ***p ≤ 0.001, ****p ≤ 0.0001.

In previous findings, the immunomodulatory impact of TRI MP-induced Tregs not only resulted in a reduction in key signs of disease, but also a decrease in pro-inflammatory cytokine expression and increase pro-regenerative cytokine expression in the local microenvironment^12,13,15^. An inspection of sample sagittal sections that were stained using Masson–Goldner (Fig. 4) qualitatively supports some preservation of the periodontal ligament space for all groups with a mix of tissue necrosis and regeneration of the gingival region where ligatures were applied specifically for Blank and TRI MP groups. Importantly, upon further quantitative assessment of local cytokines and cell markers there was a trend of a decrease in pro-inflammatory cytokines (Fig. 5a) and an increase of pro-regenerative factors upon TRI MP treatment, notably with some variability (Fig. 5b). While there were some inconsistent fluctuations in marker expression outside of these trends, it may again be due to the inherent acute nature of the ligature-induced model as a limitation of this study. As noted in the literature, the ligature-induced periodontitis model expedites disease progression and enables visible bone loss in as little as a few days^30^. Such acute inflammation aggressively expedites and increases the natural influx of lymphocytes to that site. This change in an already immunologically complex microenvironment, also influences the process of differentiation for the influx and local accumulation of lymphocytes (including T cells)^40^. Therefore, administering a preventative treatment on the same day an acutely inflamed microenvironment is developing, may detract from the effectiveness of the treatment. Instead, based on the data we have presented here, a stronger Treg induction response may be necessary (to counteract the inherent acute nature of the model and its effects) to yield more consistent trends in pro-inflammatory and pro-regenerative markers. Future studies would be needed to investigate how to further bolster the Treg induction response whether through dose escalation studies or increased dendritic cell (antigen-presenting cell) stimulation time to aid in a more robust Treg induction etc. Additionally, mechanistic studies to investigate both Treg direct action on osteoclasts vs indirect Treg action through anti-inflammatory mediators and also the potential off-target/ non-Treg mediated effects would also be important for a broader understanding of the impact of this treatment. Nevertheless, it is promising that in such complex conditions, TRI MP treatment is still able to have a detectable impact, indicating the feasibility of this approach.

Overall, findings of reduced bone loss, increase in Treg to T effector ratio and trends of decreasing pro-inflammatory factors and increasing pro-regenerative factors with TRI MP treatment, suggest that there could be potential for a Treg induction strategy for immunomodulation in the context of periodontitis. This strategy to increase the local concentration of Tregs for the regulation of inflammation-based destruction could also be used as the basis for treatment of other inflammation driven diseases upon further mechanistic studies.

## Materials and methods

### Microsphere fabrication

IL2, TGFβ and rapamycin microspheres were fabricated using an emulsion solvent evaporation method similar to previously described^11,12,14^ (details in Supplemental).

### Microsphere characterization

Size distribution of the microspheres was measured by volume impedance method performed on a Beckman Coulter Counter (Multisizer-3, Beckman Coulter, Fullerton, CA). In-vitro release studies were carried out on an end-over-end rotator at 37 °C. Scanning electron microscopy (SEM) (JEOL, JSM-6330F, Peabody, MA) was performed to assess the morphology of the microspheres (details in Supplemental).

### Disease induction model and treatment

All procedures and experiments were performed in accordance with all relevant guidelines and regulations including a protocol approved by the Institutional Animal Care and Use Committee at the University of Pittsburgh (protocol number: 20077447) and the ARRIVE guidelines for preclinical studies. Mice were randomly assigned to experimental groups. A 6-0 silk suture (Henry Schein, Melville, NY) was tied around the left maxillary second, similar to as previously described^30^. Treatment injections were administered on the same day. Ligatures were left in place for 7 days before sacrifice (details in Supplemental).

### Alveolar bone loss analysis

Micro-computed tomography (uCT) was used to assess bone loss (Scanco uCT 50, Scanco Medical, Switzerland). Blinded measurements of the distance between the cementoenamel junction (CEJ) of the maxillary second molar and the alveolar bone crest (ABC) at mesial and distal aspects were performed (details in Supplemental).

### Histological analysis

Demineralized maxillae embedded in paraffin were cut into sagittal sections 5 µm thick. For tissue and bone contrast, tissue sections were stained with Masson–Goldner according to the manufacturer instructions. For immunofluorescence, samples were stained with primary antibodies; biotin-FoxP3 (FJK-16s; eBio) and CD3 (SP7, monoclonal rabbit IgG; Thermo Scientific, Waltham, MA) and secondary antibodies Cy3-streptavidin (Jackson ImmunoResearch Laboratories, West Grove PA), Alexa Fluor 555 donkey anti-rabbit IgG (Thermo Scientific) and DAPI, similar to as previously described^13^. Samples were imaged with a fluorescent microscope (Eclipse TE200-E; Nikon Instruments) and a blinded quantification was performed of the ratio of FOXP3 positive cells (Tregs) to CD3 positive cells (total T cells) (details in Supplemental).

### Quantitative polymerase chain reaction (qPCR)

To analyze gene expression, RNA was extracted from resected maxilla gingival tissue with Trizol reagent (Life Technologies), purified using RNeasy Mini Kit (Qiagen) and converted to cDNA using a High-Capacity RNA-to-cDNA Kit (Applied Biosystems). Reactions were run on a QuantStudio 6 Flex Real-Time PCR system (Thermo Fisher Scientific) and the delta-delta Ct method was used for analysis (details in Supplemental).

### Statistics

Statistical analyses were performed using GraphPad Prism Software v9. A One-way ANOVA with a Tukey post-hoc test was used to compare the mean of each experimental group with the mean of every other group. To estimate sample size, a power level of 0.8 and an alpha of 0.005 were used (details in Supplemental).

## Supplementary Information

Supplementary Information.
