# Supplementary material for: Local induction of regulatory T cells prevents inflammatory bone loss in ligature-induced experimental periodontitis in mice
Source: Sci Rep. 2022 Mar 23;12:5032. doi: 10.1038/s41598-022-09150-8 (PMC8943171; doi:10.1038/s41598-022-09150-8)
Supplement: Supplementary file 1 — Supplementary Information. [file 41598_2022_9150_MOESM1_ESM.pdf]

Local Induction of Regulatory T cells Prevents Inflammatory Bone Loss in Ligature-Induced Experimental Periodontitis in Mice

Ashlee C. Greene[1], Mostafa Shehabeldin[2], Jin Gao[3], Stephen C. Balmert[4], Michelle Ratay[6], Charles Sfeir[2,3,5,7], Steven R. Little*[1,6,7,8,9,10]

[1]Department of Chemical and Petroleum Engineering, University of Pittsburgh, Pittsburgh, PA, USA

[2]Department of Oral and Craniofacial Sciences, School of Dental Medicine, University of Pittsburgh, Pittsburgh, PA, USA

[3]Department of Periodontics and Preventative Dentistry, School of Dental Medicine, University of Pittsburgh, Pittsburgh, PA, USA

[4]Department of Dermatology, University of Pittsburgh, Pittsburgh, PA, USA

[5]Center for Craniofacial Regeneration, University of Pittsburgh, Pittsburgh, PA, USA

[6]Department of Bioengineering, University of Pittsburgh, Pittsburgh, PA, USA

[7]McGowan Institute for Regenerative Medicine, University of Pittsburgh, Pittsburgh, PA, USA

[8]Department of Immunology, University of Pittsburgh, Pittsburgh, PA, USA

[9]Department of Ophthalmology, University of Pittsburgh, Pittsburgh, PA, USA

[10]Department of Pharmaceutical Sciences, University of Pittsburgh, Pittsburgh, PA, USA

*Corresponding Author:

Steve R. Little, PhD
Department of Chemical and Petroleum Engineering
University of Pittsburgh, Pittsburgh, PA, USA
Email: srlittle@pitt.edu

Materials and Methods

Microsphere fabrication

For IL-2 microspheres, a water-oil-water double emulsion was used. Two hundred (200) mg of acid terminated Poly (lactic-co-glycolic) acid (PLGA) (RG502H; 50:50 lactide:glycolide, MW: 7-17kDa, Sigma Aldrich) was dissolved in 4mL of dichloromethane (DCM). For the 200uL inner aqueous phase, 5ug of recombinant mouse IL-2 (R&D Systems, Minneapolis, MN) aliquoted in 50uL of phosphate buffered saline without calcium or magnesium (PBS (-)) was added to 150uL of PBS (-). The first water-in-oil emulsion was prepared by sonicating the 200uL inner aqueous solution of IL-2 in the 4mL of dissolved polymer at 55% amplitude for 10s (Active Motif, Carlsbad, CA). The resulting primary emulsion is then poured into 60mL of 2% w/v Poly (vinyl alcohol) (PVA, MW ~25 kDa, 98% hydrolyzed, Polysciences, Warrington, PA) with 51.6mM NaCl and homogenized at 3,000rpm for 1min (L4RT-1, Silverson, East Longmeadow, MA). The resulting double emulsion was then poured into 80mL of 1% w/v PVA with 51.6mM NaCl and with a stir bar and stir plate, stirred on ice at 600rpm for 3hrs to allow for the DCM to evaporate.

For TGF-β microspheres, similarly, 170mg of ester terminated Poly (lactic-co-glycolic) acid (PLGA) (RG502; 50:50 lactide:glycolide, MW: 7-17kDa, Sigma Aldrich) and 30mg of mPEG-PLGA (50:50 lactide:glycolide, 5-20 kDa, PolySciTech, West Lafayette, IN) were dissolved in 4mL of dichloromethane (DCM). For the 200uL inner aqueous phase, 10ug of recombinant human TGF-β (Peprotech, Rocky Hill, NJ) aliquoted in 100uL of deionized (DI) water was added to 100uL of DI water. The first water-in-oil emulsion was prepared by sonicating the 200uL inner aqueous solution of TGF-β in the 4mL of dissolved polymer at 55% amplitude for 10s (Active Motif, Carlsbad, CA). The resulting primary emulsion is then poured into 60mL of 2% w/v PVA in DI water and homogenized at 3,000rpm for 1min. The resulting double emulsion was then poured into 80mL of 1% w/v PVA in DI water and, with a stir bar and stir plate, stirred on ice at 600rpm for 3hrs to allow for the DCM to evaporate.

For rapamycin microspheres, an oil-water single emulsion was used. Two hundred (200) mg of acid terminated Poly (lactic-co-glycolic) acid (PLGA) (RG502H; 50:50 lactide:glycolide, MW: 7-17kDa, Sigma Aldrich) was dissolved in 4mL of dichloromethane (DCM). Next, the polymer solution was poured into 60mL of 2% w/v Poly (vinyl alcohol) (PVA, MW ~25 kDa, 98% hydrolyzed, Polysciences, Warrington, PA) in DI water and homogenized at 3,000rpm for 1min (L4RT-1, Silverson, East Longmeadow, MA). The resulting single emulsion was then poured into 80mL of 1% w/v PVA in DI water and, with a stir bar and stir plate, stirred on ice at room temperature for 3hrs to allow for the DCM to evaporate.

For all microspheres, a polymer only control microsphere (blank) was also prepared. After stirring to allow for solvent evaporation, all microspheres were collected by centrifugation (200g, 5min, 4C) and washed 4 times with DI water. In a final suspension of >5mL DI water, samples were then flash frozen by liquid nitrogen and lyophilized for at least 48 hours (Virtis Benchtop K freeze dryer, Gardiner, NY; operating at 100mTorr).

Microsphere characterization

Sizing- Size distribution of the microspheres was measured by volume impedance method performed on a Beckman Coulter Counter (Multisizer-3, Beckman Coulter, Fullerton, CA). Approximately 1 mg of particles were suspended in 20mL of Isoton II diluent solution (Beckman Coulter) and readings were taken. Samples were diluted as necessary. A particle count of at least 1,000 was used to obtain number and volume averaged sizes.

In vitro release studies were carried out by incubating 10mg of microspheres placed in 1mL PBS (without calcium, magnesium) (HyClone, Cytiva, Marlborough, MA) with either 1% w/v bovine serum albumin (BSA) (for TGFB and IL2 MPs) or 0.2% Tween-80 (for Rapamycin MPs) on an end-over-end rotator at 37°C. At each time point, samples were centrifuged at 22°C at 371rcf for 5min and 800uL of supernatant was collected and replaced. Supernatant samples were quantified using an enzyme-linked immunosorbent assay (ELISA) (R&D Systems, Minneapolis, MN). For rapamycin MPs, cumulative release was quantified by UV-vis spectroscopy on a microplate reader (absorbance at 278nm).

Scanning electron microscopy (SEM) (JEOL, JSM-6330F, Peabody, MA) at 3kV, 8-8.5mm WD and x2000 magnification was performed to assess the morphology of the microspheres. Dry powder samples were mounted onto a stub with copper tape and sputter coated with palladium prior to imaging.

Disease induction Model and Treatment

Male Balb/c wild type mice aged 6-8weeks old were purchased from Charles River (Wilmington, MA). All procedures were performed in accordance with a protocol approved by the Institutional Animal Care and Use Committee at the University of Pittsburgh (protocol number: 20077447). First, mice were randomly assigned to experimental groups; age control, diseased untreated (ligature only), vehicle control (ligature + blank MPs) and treated (ligature + TRI MPs). Next, for disease induction, mice were anesthetized by I.P. injection of Ketamine/Xylazine. Mice were fed a standard pellet diet and water through the course of the study. A 6-0 silk suture (Henry Schein, Melville, NY) was then tied around the left maxillary second molars using a stereomicroscope, similar to as previously described[17]. Following ligature placement, microspheres (either Blank MPs or TRI MPs), at 20mg MPs/mL suspended in 2% carboxymethylcellulose in PBS (to help ensure a longer residence time) were locally injected. An injection of 30 uL was distributed along the palatal side and an additional 20 uL injection was placed on the buccal side of the ligated maxillary second molar. Ligatures were left in place for 7 days before sacrifice (no ligatures were lost during the course of these studies).

Alveolar Bone loss analysis

Micro-computed tomography (uCT) was used to assess bone loss. Mice maxillae were first harvested and fixed in 10% buffered formalin overnight prior to being transferred to 70% ethanol for uCT scanning with a resolution of 10 μm voxel size (Scanco uCT 50, Scanco Medical, Switzerland). Scans were then reoriented using DataViewer software (GE Healthcare) to a standardized orientation based on predetermined anatomical guides. CTAN software (Bruker,

Massachusetts) was then used to perform blinded measurements of the distance between the cementoenamel junction (CEJ) of the maxillary second molar and the alveolar bone crest (ABC) at mesial and distal aspects. A total of 8 measurement slices with 40 μm in between were used for each sample. Results of the distances from the ligated side were normalized to the corresponding average from the healthy, right side of the same maxillae.

Histological Analysis

To prepare for histological analysis, mice maxillae were demineralized in 10% EDTA solution for 2 weeks. Samples were then embedded in paraffin and cut into sagittal sections 5 μm thick for immunohistochemistry and immunofluorescence staining. For tissue and bone contrast, tissue sections were stained with Masson-Goldner according to the manufacturer instructions. Samples we qualitatively assessed for bone loss and changes in gingiva and periodontal ligament for comparison. To determine changes in total T cells and FOXP3 T cells after TRI administration, samples were first deparaffinized followed by an antigen retrieval step using 10mM pH 6 Na Citrate with 0.05% Tween 20. Next, an autofluorescence reduction step was performed using 10mM Copper Sulfate and 50mM NH4 Acetate. Sections were then blocked with PBS containing 5% donkey serum and 1% Tween20 and treated with a streptavidin/biotin blocking kit (Vector Labs, Burlingame, CA). The blocked sections were then incubated overnight at 4°C with primary antibodies; biotin-FoxP3 (FJK-16s; eBio) and CD3 (SP7, monoclonal rabbit IgG; Thermo Scientific, Waltham, MA). Sections were then incubated with secondary antibodies Cy3-streptavidin (Jackson ImmunoResearch Laboratories, West Grove PA) and Alexa Fluor 555 donkey anti-rabbit IgG (Thermo Scientific) for 1 hr at room temperature, counter stained with DAPI and fixed with 2% paraformaldehyde, similar to as previously described[13]. Samples were imaged with a fluorescent microscope (Eclipse TE200-E; Nikon Instruments) and a blinded quantification was performed of the ratio of FOXP3 positive cells (Tregs) to CD3 positive cells (total T cells).

Quantitative Polymerase Chain Reaction (qPCR)

To analyze gene expression, maxillae were flash frozen in liquid nitrogen for storage at -80C until RNA extraction. The day before RNA extraction, each sample was thawed in RNA later solution at 4°C overnight. Then, gingival tissue was resected from the ligated side of the maxilla under a dissection microscope and Trizol reagent (Life Technologies) was used to extract total RNA from the gingiva samples. Purification was performed using RNeasy Mini Kit (Qiagen). For each reverse transcription reaction, 450ng of RNA was converted to cDNA using a High-Capacity RNA-to-cDNA Kit (Applied Biosystems). Quantitative polymerase chain reaction was then performed using TaqMan Gene Expression Master Mix and markers specific for TNFSF11 (Mm00441906_m1; FAM-MGB), NCF1 (Mm00447921_m1; FAM-MGB), IFNG (Mm01168134_m1; FAM-MGB), IL4 (Mm00432102_m1; FAM-MGB), TGFβ (Mm01178820_m1; FAM-MGB), IL10 (Mm01288386_m1; FAM-MGB), COL1A1(Mm00801666_g1; FAM-MGB), TIMP1(Mm01341361_m1; FAM-MGB) and GAPDH as the endogenous control (Mm99999915_g1; FAM-MGB). Reactions were run on a QuantStudio 6 Flex Real-Time PCR system (Thermo Fisher Scientific) and the delta-delta Ct method was used for analysis.

Statistics

Sample size was determined based on previously reported results and preliminary data. G*Power 3.1 software with a power level of 0.8 and alpha of 0.05 were used in estimating sample size. Statistical analyses were performed using GraphPad Prism Software v9. A One-way ANOVA with a Tukey post-hoc test was used to compare the mean of each experimental group with the mean of every other group. A ROUT outlier test with a threshold for outlier removal (Q = 0.1%) was used to remove outliers from the graph of average interdental bone loss. Data are expressed as a scatter plot with the mean or bar graph with the mean ± standard deviation. The following cutoffs were used for statistical significance; * $p \leq 0.05$, ** $p \leq 0.01$, *** $p \leq 0.001$, **** $p \leq 0.0001$.

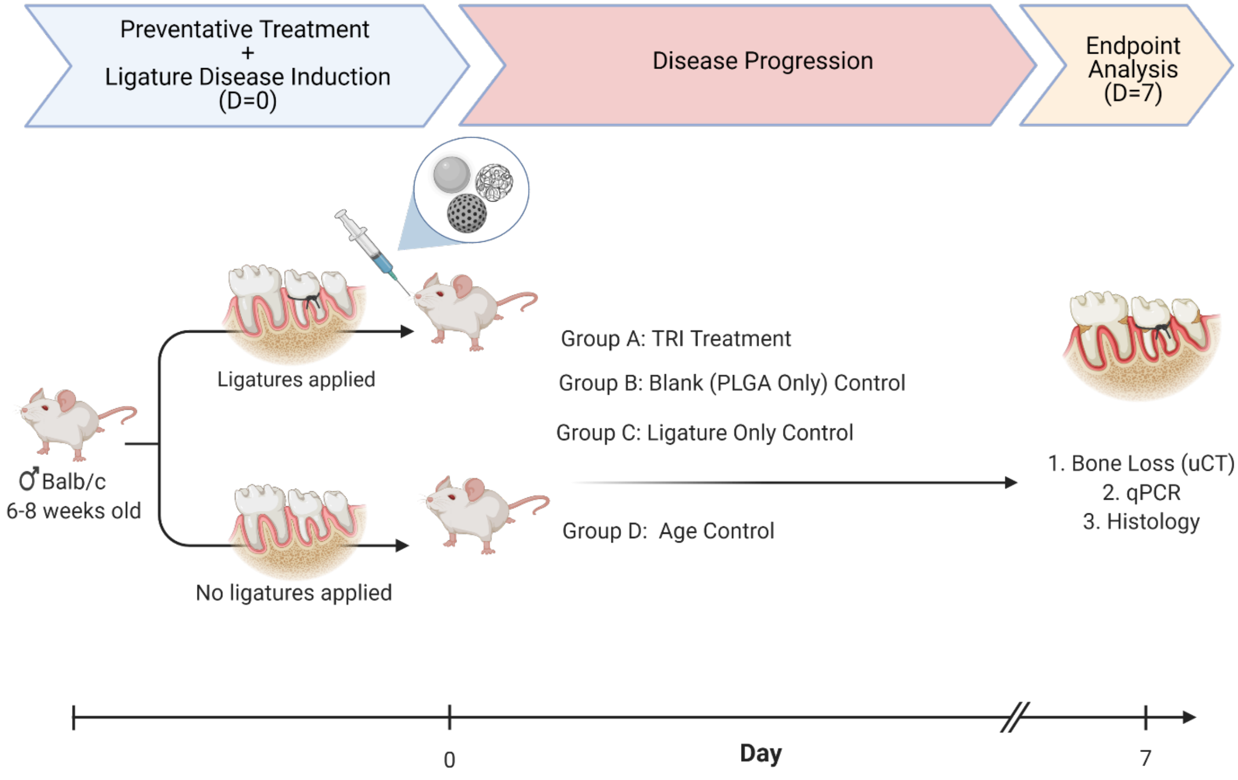

Supplementary Figure S1. Murine Ligature-Induced Periodontal Disease Timeline. Experimental design where male Balb/c mice (6-8 weeks old) on Day 0 are either in; Group A-ligatures applied with a TRI microsphere injection, Group B- ligatures applied with blank PLGA (vehicle only) microsphere injection, Group C- ligatures applied only or Group D- no ligatures applied (age control). On Day 7, samples were collected to quantify bone loss by micro-CT (uCT), qPCR and histology as endpoint analyses.
